# Topical Sustained Delivery of Miltefosine Via Drug-Eluting Contact Lenses to Treat Acanthamoeba Keratitis

**DOI:** 10.3390/pharmaceutics14122750

**Published:** 2022-12-08

**Authors:** Lin Chen, Liangju Kuang, Amy E. Ross, Wissam Farhat, Nikolay Boychev, Sina Sharfi, Levi N. Kanu, Longqian Liu, Daniel S. Kohane, Joseph B. Ciolino

**Affiliations:** 1 Department of Optometry and Visual Science, West China Hospital, Sichuan University, Chengdu 610041, China; 2 Department of Ophthalmology, Affiliated Hospital of Zunyi Medical University, Zunyi 563000, China; 3 Department of Ophthalmology, Schepens Eye Research Institute of Massachusetts Eye and Ear Infirmary, Harvard Medical School, Boston, MA 02114, USA; 4 Department of Anesthesia, Boston Children’s Hospital, Harvard Medical School, Boston, MA 02115, USA

**Keywords:** acanthamoeba keratitis, controlled drug delivery, contact lens, miltefosine, PLGA

## Abstract

This study aimed to develop a miltefosine-eluting contact lens (MLF-CL) device that would allow sustained and localized miltefosine release for the treatment of Acanthamoeba keratitis. MLF-CLs were produced in three different miltefosine doses by solvent-casting a thin miltefosine-polymer film around the periphery of a methafilcon hydrogel, which was then lathed into a contact lens. During seven days of in vitro testing, all three formulations demonstrated sustained release from the lens at theoretically therapeutic levels. Based on the physicochemical characterization of MLF-CLs, MLF-CL’s physical properties are not significantly different from commercial contact lenses in terms of light transmittance, water content and wettability. MLF-CLs possessed a slight reduction in compression modulus that was attributed to the inclusion of polymer-drug films but still remain within the optimal range of soft contact lenses. In cytotoxicity studies, MLF-CL indicated up to 91% viability, which decreased proportionally as miltefosine loading increased. A three-day biocompatibility test on New Zealand White rabbits revealed no impact of MLF-CLs on the corneal tissue. The MLF-CLs provided sustained in vitro release of miltefosine for a week while maintaining comparable physical features to a commercial contact lens. MLF-CL has a promising potential to be used as a successful treatment method for Acanthamoeba keratitis.

## 1. Introduction

Acanthamoeba keratitis (AK) is a rare but potentially blinding corneal infection caused by free-living Acanthamoeba amoebae [1,2]. Inadequate disinfection of commercial contact lenses is one of the most common risks of AK [3,4,5]. With the increasing number of people wearing contact lenses, the prevalence of Acanthamoeba infections in the world is on the rise [6,7,8,9,10]. AK treatment at present is suboptimal and the prognosis of this condition is often poor [11]. First-line therapies for AK include topical polyhexamethylene biguanide (PHMB), propamidine isethionate, chlorhexidine, dibromopropamidine, and/or hexamidine [12,13], often requiring two or more drugs. However, the treatment duration is lengthy, complicated, and is often ultimately unsuccessful in complete eradication. Frequent eye drop instillation, as often as hourly throughout the day [14] is used in clinical practice, in part because of the limited bioavailability of topical eye drops [15]. Bioavailability from eye drops is estimated to be less than 5% because of the anatomic barriers in the anterior segment of the eye (e.g., tear film barrier and corneal barrier) [16,17]. In addition, trophozoites and cysts (two forms of Acanthamoeba’s lifecycle) can remain in the cornea for over two years [2,18]. Non-adherence can lead to suboptimal dosing. In general, killing of amoebic trophozoites and cysts is a challenge.

Miltefosine is an alkylphosphocholine agent with a broad spectrum of antiparasitic properties, which has recently been used in AK. This drug is commonly used as an oral medication to treat leishmaniasis [19]. After obtaining orphan drug designation from the FDA in 2016, oral miltefosine has been shown to be effective in refractory AK in several recent case reports [20,21,22,23]. However, systemic administration can produce severe inflammatory responses [24,25]. Providing a topical treatment could mitigate or even eliminate miltefosine’s systemic side effects. Although miltefosine eye drops were effective in the management of AK in hamster [26] and rat [27] models, a clinical study failed to replicate the results in the humans [28]. The ineffectiveness observed may be attributed to the low bioavailability of eye drops in general and the fact that all studied cases had advanced keratitis at the time of initiation of the topical miltefosine therapy.

Previously, we have described a contact lens drug delivery system involving the incorporation of a thin drug-poly[lactic-co-glycolic acid] (PLGA) film into a poly[hydroxyethyl methacrylate] (pHEMA) hydrogel. This system has previously been used to achieve zero-order release kinetics at therapeutically relevant concentrations with fluorescein and ciprofloxacin for up to one month [29].

In this study, we incorporated miltefosine into this contact lens system to create a therapeutic contact lens (MLF-CL) for the treatment of AK. Miltefosine was loaded at different concentrations and the physicomechanical properties (hydration, water contact angle, mechanical strength) of the MLF-CLs were investigated and compared with commercial contact lenses. Drug release kinetics were measured for a period of one week. In addition, the cytocompatibility and in vivo biocompatibility of the MLF-CL was evaluated.

## 2. Materials and Methods

### 2.1. Materials

Miltefosine was purchased from Cayman Chemical (Ann Arbor, MI, USA). Liquid methafilcon and methafilcon contact lens blanks were purchased from Kontur Kontact Lens (Hercules, CA, USA). Poly(lactide-co-glycolide), 85% lactide, 15% glycolide (MW 190 KDa) was purchased from Merck and Cie (Schaffhausen, Switzerland). Hexafluoroisopropanol (HFIP) was purchased from Oakwood Chemical (Estill, NC, USA). Gibco phosphate-buffered saline (PBS), Dulbecco’s Modification of Eagle’s Medium, penicillin-streptomycin, and L-glutamine were purchased from Thermo Fisher Scientific (Waltham, MA, USA). Biopsy punches were purchased from Sklar Instruments (Westchester, PA, USA). All other reagents were purchased from Millipore-Sigma (St. Louis, MO, USA), except where otherwise noted.

### 2.2. MLF-CL Fabrication

**Scheme 1:**
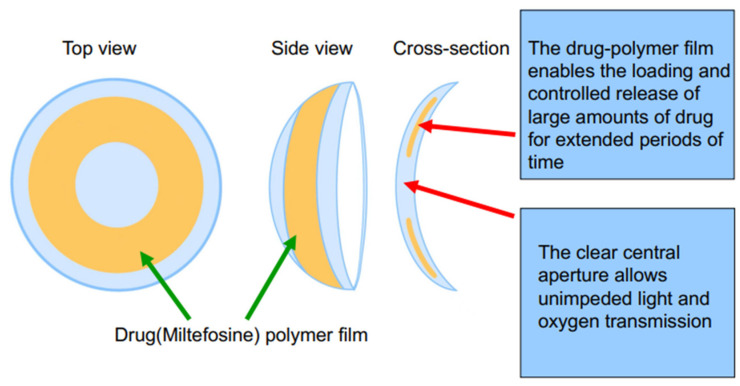
Schematic illustration of the designed miltefosine-eluting contact lens (MLF-CL).

The drug-polymer solution was created by dissolving miltefosine and PLGA in HFIP, in ratios provided in Table 1. The polymer concentration was kept at 50 mg/mL. The film was created by using solvent casting. 50 µL of solution was then pipetted into a concavity lathed into a cylinder of dry polymerized methafilcon After 6 min of rotation on a spin coater (Model SC100, Best Tools LLC, St. Louis, MO, USA), the liquid HFIP evaporated, leaving a drug-polymer film. A 4 mm biopsy punch was used to define and remove the central diameter of the drug-polymer film to create a clear central aperture. The hydrogel blanks containing the films were placed on a desiccator under vacuum for two weeks to remove residual HFIP. The side of the film that was not yet in contact with methafilcon was then encapsulated in methafilcon through ultraviolet (UV) photopolymerization using ethylene glycol dimethacrylate (0.3%, crosslinker), Irgacure^®^ 2959 (9 mg/mL, photoinitiator) and 3.5 min of UV light exposure at 120 mW/cm^2^ applied within a Zeta 7401 UV chamber (Loctite, Düsseldorf, Germany) for 3.5 min at 120 mW/cm^2^. The methafilcon cylinder was then lathed into a contact lens that consisted of the drug-PLGA film fully encapsulated within methafilcon, with the initial cylinder on the front curve and polymerized methafilcon on the back curve (Scheme 1).

### 2.3. Optical Coherence Topography

To evaluate MLF-CL morphology and film thickness, all formulations were imaged using anterior segment optical coherence topography (AS-OCT, RTVue, Optovue, Fremont, CA, USA) to assess the morphology. Non-hydrated MLF-CLs were positioned with the convex side of the lens facing the OCT camera. Raster scanning imaging was used in four segments for each contact lens to obtain cross sectional images of the contact lens and drug-polymer film.

### 2.4. MLF-CL Release

In vitro release of the MLF-CLs was conducted by hydrating MLF-CLs in 5 mL phosphate buffered saline (PBS) and placing in an incubator shaker at 37 °C at 64 rpm. At predetermined time points, the MLF-CLs were removed from PBS and immersed in fresh PBS. Release buffer aliquots were stored at 4 °C until drug concentration was quantified. Four lenses were used per formulation. Release quantification was performed by a colorimetric method.

### 2.5. Water Content

Water content of the MLF-CLs was performed by gravimetric method, according to ISO-18369-1 guidelines [30]. MLF-CLs were immersed in PBS until reaching equilibrium swelling, defined by a less than 5% change in mass in a 24 h period. Weight was recorded, then MLF-CLs were dried at 60 °C overnight and re-weighed. Weights were taken immediately after removal from the oven to prevent atmospheric moisture from influencing the results. All weights were recorded in duplicate. Four lenses were used per formulation. Percent water content was calculated by the following formula

$$\frac{W_{s}-W_{d}}{W_{s}}\times 100\%$$

where *W_s_* = weight at equilibrium swelling and *W_d_* = dry weight.

### 2.6. Light Transmission

Light transmission was measured through commercial contact lenses and the central aperture of the hydrated MLF-CL using SpectraMax M3 [31]. The average light transmission was calculated by averaging the transmission over the visible light spectrum (390–700 nm).

### 2.7. Water Contact Angle

MLF-CLs were hydrated in PBS until they reached equilibrium swelling. A drop of distilled water (about 5 µL) onto the surface via a syringe. Then, a high-resolution image was captured from the side using the Dino-Lite Edge camera. The contact angle for each formulation (*n* = 4 and 5 measurements per specimen) was determined using ImageJ software (NIH, Bethesda, MD, USA) as a function of time.

### 2.8. Mechanical Properties

The mechanical features of the MLF-CLs were determined at room temperature using a Mark-10 ESM 303 motorized test stand (Mark-10 Corporation, Copiague, NY, USA). Before analysis, samples were hydrated by immersing them in PBS for 2 days. The compression test was conducted on rings with 7 mm and 6 mm external and internal diameter, respectively and with a crosshead speed of 1 mm/min. The Compression Modulus was obtained from the linear derivative of the stress–strain curve in the low stiffness range (less than 10% strain). Three independent measurements were conducted for each group.

### 2.9. Cytotoxicity

L929 murine fibroblasts (American Tissue Culture Collection, Manassas, VA, USA) were used to assess the cytotoxicity of MLF-CLs. L929 cells were grown in Dulbecco’s Modification of Eagle’s Medium (DMEM), supplemented with 10% fetal bovine serum, 1% penicillin-streptomycin, and 4 mM L-glutamine. Cytotoxicity was measured by the minimum elution media method, described by ISO 10993-5 guidelines [32]. MLF-CLs or vehicle lenses (with the polymer film but no drug) were immersed in supplemented DMEM in a volume of 1.8 mL for each MLF-CL. The MLF-CLs were incubated at 37 °C for 24 h to capture eluted drugs and leachables. The elution media (extract) was applied neat to L929s plated in 96 well plates at 1 × 10^5^ cells/mL the day prior. The cells with extract were incubated for 24 h. Cell viability was measured by CellTiter 96^®^ Non-Radioactive Cell Proliferation Assay (MTT) (Promega, Madison, WI, USA). Results were expressed as percentage viability of L929 controls that were not exposed to extract. Four lenses were used for each formulation, with six replicates per lens.

### 2.10. Animals

The study protocol #2021N000159 was approved on 7 July 2021 by the Institutional Animal Care and Utilization Committee at Schepens Eye Research Institute of Massachusetts Eye and Ear Infirmary (Boston, MA). All animals were treated according to the Association for Research in Vision and Ophthalmology (ARVO) Statement for the Use of Animals in Ophthalmic and Vision Research (ARVO Handbook, 1993). New Zealand white rabbits (NZW) (Charles River Laboratories, Boston, MA, USA) age four months, each weighing 3–5 kg, were used for the biocompatibility study. Only one eye in each NZW was used to prevent bilateral vision restriction. The contralateral eye was untreated and served as a control. NZWs were singly housed in a climate-controlled environment with free access to food and water during acclimation and throughout the experiment. NZWs were acclimated for seven days prior to experiments. In each of the studies, intramuscular injection of 30 mg/kg ketamine, 5 mg/kg xylazine, and 1 mg/kg acepromazine were used for anesthesia. 0.05 mg/kg Buprenorphine HCl was used for pain control prior to tarsorrhaphy.

Only MLF-CL_4_ was used for the biocompatibility study. Under anesthesia, NZWs had baseline photos taken by a Topcon DC-3 digital camera attachment for slit lamp. The MLF-CL was placed on the cornea, with care to place it under the nictating membrane. The MLF-CL was evaluated to ensure free movement on the cornea without fluting or bubbling. To improve lens retention, eyes with contact lenses received a temporary tarsorrhaphy in which the eyelids were closed with one to two 5-0 Nylon sutures. NZWs were evaluated daily for signs of discomfort. After three days, the tarsorrhaphy and MLF-CL and re-imaged. NZW eyes were evaluated for redness, edema, discharge, and fur matting. NZWs were euthanized with 120 mg/kg pentobarbital, and the corneas were removed from both eyes. The eyes were fixed in 10% formalin, embedded in paraffin, and cut into 10-micron sections in an anterior to posterior fashion so that the pupil and the optic nerve were in one section (PO section). Slides were stained with hematoxylin and eosin (H&E) to visualize ocular structures.

### 2.11. Statistics

For all continuous data, nonparametric descriptive statistics were used to determine statistical significance. Kruskal–Wallis Test was calculated, and if significant, followed by pairwise comparisons to determine significant differences between groups. A *p*-Value of less than 0.05 was considered significant.

## 3. Results

### 3.1. MLF-CL Was Successfully Encapsulated in Contact Lens Hydrogels

**Figure 1:**
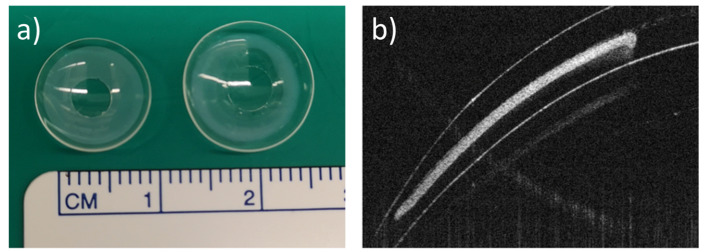
Miltefosine-eluting contact lens (MLF-CL). (**a**) Photographs of dry (left) and hydrated (right) contact lens. (**b**) An ocular coherence tomography (OCT) image of a section of a dry MLF-CL showing an encapsulated miltefosine-polymer film and central aperture to allow for unimpeded vision.

Three dosages of MLF-CLs were created by changing the drug amount loaded in each lens while keeping PLGA concentration constant (Table 1). Drug-polymer (PLGA) films were created through solvent casting, then encapsulated in methalfilcon hydrogel using ultraviolet (UV) polymerization of hydroxyethylmethacrylate (HEMA) (Figure 1). When hydrated, the MLF-CLs expanded from 11.3 mm to 14.5 mm diameter and from 4 mm to 5 mm central aperture diameter and maintained a normal shape (Figure 1a). Anterior segment ocular coherence tomography (AS-OCT) images demonstrated uniform drug-polymer films completely within the methafilcon hydrogel and a clear aperture to preserve light transmission to the visual system (Figure 1b). Film thickness was roughly the same for all groups: MLF-CL_2_: 83.30 ± 6.08 μm, MLF-CL_4_: 81.11 ± 1.15 μm, MLF-CL_8_: 85.03 ± 5.62 μm) (*p* = 0.779, Kruskal–Wallis, *n* = 4). When imaged by OCT, no delamination of the film from the hydrogel was observed before hydration or after the lens was hydrated (Figure S1), indicating the film had sufficient adherence to the hydrogel that allow the film to maintain the same configuration during the hydration process which increased the diameter of the lens.

**Figure 2:**
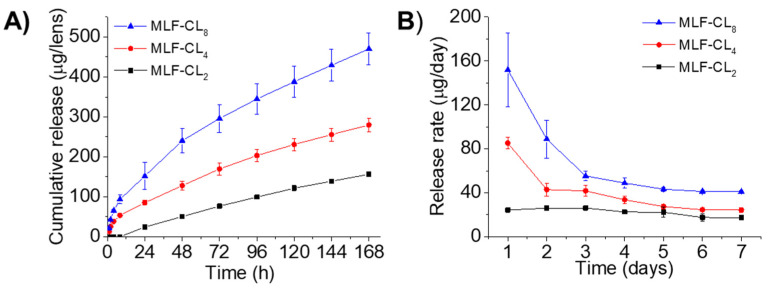
In vitro miltefosine (MLF) release from MLF-CLs. (**A**) the cumulative release and (**B**) the daily release rate. Quantitative data are expressed as mean ± standard deviation (SD), *n* = 4/group per time point.

Drug release from MLF-CLs was studied in PBS at 37 °C. Because miltefosine aqueous solubility is relatively high (reported as 440 mg/mL [33]), frequent buffer changes were performed to preserve infinite sink conditions. All formulations demonstrated relatively constant state release through seven days (Figure 2). Cumulative 7-day miltefosine release ranged from 156.0 ± 4.7 µg (MLF-CL_2_) to 470.3 ± 39.8 µg (MLF-CL_8_) (Figure 2). We analyzed the miltefosine release kinetics from the MLF-CL using zero-order, first-order, Higuchi, and Korsmeyer-Peppas mathematical models (Table S1). Considering the highest value of R^2^-adjusted, the lowest Akaike Information Criterion (AIC) values and the largest Model Selection Criterion (MSC) [34], the release data fit well to the Korsmeyer-Peppas model for all formulations (Figure S2 and Table S1). The predicted release based on Korsmeyer-Peppas model was shown in Figure S2. These results indicated the diffusional release of miltefosine from contact lens, which was consistent with other drugs released from therapeutic contact lenses [31,35].

### 3.2. MLF-CL Water Content Was Comparable to Commercial Contact Lenses

**Figure 3:**
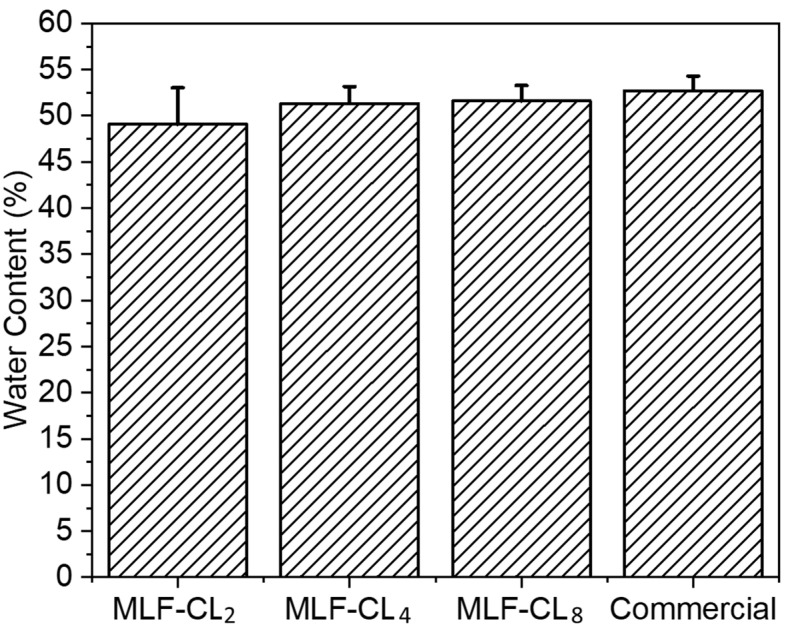
Equilibrium water content for miltefosine-eluting contact lenses (MLF-CLs) compared to commercial contact lenses. Quantitative data are expressed as mean ± standard deviation (SD). No significant statistical difference among samples by Kruskal–Wallis Test. *p* = 0.49, *n* = 4.

The water content of contact lenses is reported to have a significant impact on oxygen permeability and contact lens wearing comfort [36,37]. MLF-CL water content was assessed by the gravimetric method [38] and the results were compared to commercial methafilcon contact lenses (Kontur) (*n* = 4/group). Commercial contact lens had a water content (53 ± 1.6%) comparable to the reported value (55%) [39,40]. There was no significant (*p* = 0.49, Kruskal–Wallis Test) difference in the water content between MLF-CLs and commercial methafilcon lenses (Figure 3).

### 3.3. MLF-CL Light Transmittance Was comparable to Commercial Contact Lenses

**Figure 4:**
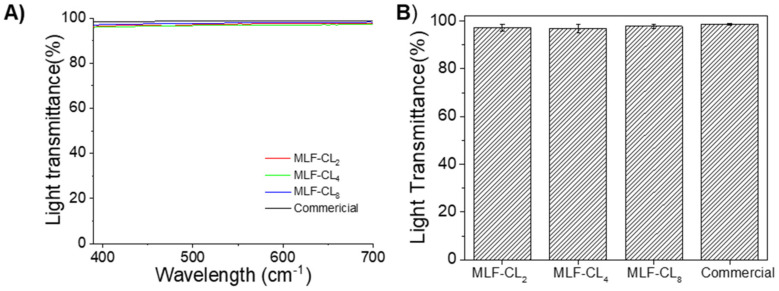
(**A**) Light transmittance curve of commercial lens and central aperture of MLF-CL through the visible light spectrum (from 390 nm to 700 nm). (**B**) The average light transmission of MLF-CL, which was calculated by averaging the transmission over the visible light spectrum (390–700 nm). *n* = 4 lens per group. Quantitative data are expressed as mean ± standard deviation (SD). No significant difference in average light transmittance between the commercial contact lenses and MLF-CL was observed (*p* = 0.15, Kruskal–Wallis Test).

The light transmittance properties of contact lenses are clearly important for visual performance. MLF-CL maintained light transmittance >95% through the central aperture between 390 nm to 700 nm (Figure 4A). The difference in average light transmittance between the commercial contact lenses and MLF-CL was negligible (*p* = 0.15, Kruskal–Wallis Test, *n* = 4 per group, Figure 4B), which was in agreement with our previous reports [35,41].

### 3.4. Compression Modulus Was Similar between MLF-CLs and Commercial Contact Lenses

**Figure 5:**
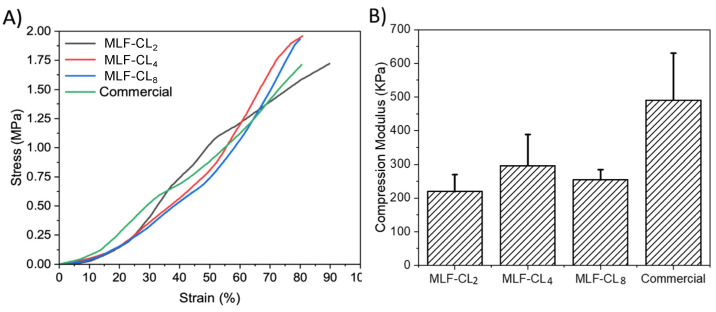
(**A**) Average stress–strain curves and (**B**) Compression modulus for miltefosine-eluting contact lenses (MLF-CLs) compared to commercial contact lenses. Quantitative data are expressed as mean ± standard deviation (SD). No significant statistical difference among samples. by Kruskal–Wallis Test. *p* = 0.1, *n* = 3.

Excessive stiffness in a contact lens can result in discomfort. Compression tests [42] were performed on the various MLF-CLs formulations to estimate their mechanical properties. The stress–strain curves of the different lenses were plotted, hence providing a visual display, indicating their strength and elasticity. MLF-CLs were compared to commercial CLs (*n* = 3/group, Figure 5). All groups were compared by Kruskal–Wallis Test. Commercial CLs demonstrated the highest modulus at 491 ± 140 kPa. MLF-CL_2_ (220 ± 50 kPa), MLF-CL_4_ (296 ± 92 kPa) and MLF-CL_8_ (255 ± 30 kPa) with no significant differences among them (*p* = 0.1, Kruskal–Wallis Test).

### 3.5. MLF-CL Surface Wettability was Comparable to Commercial Contact Lenses

**Figure 6:**
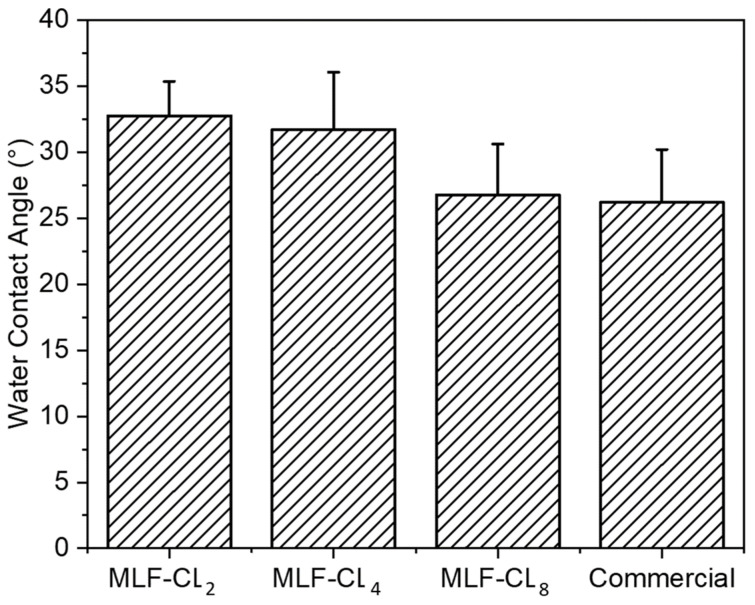
Water contact angle of miltefosine-eluting contact lenses (MLF-CLs) and commercial contact lenses. Quantitative data are expressed as mean ± standard deviation (SD). No significant statistical difference among samples by Kruskal–Wallis Test. *p* = 0.06, *n* = 4.

Water contact angle is a measure of the wettability of a surface. Poor wettability can break up the tear film, leading to dry eyes [36,43]. Water contact angle was measured in MLF-CLs and commercial contact lenses using sessile drop technique, the most commonly used method for biomaterials [44]. All contact lenses were cut to lie flat to ensure the most accurate measurements. Compared to commercial contact lenses (26.2 ± 4.0°), there were no significant differences (*p* = 0.06, Kruskal–Wallis Test) among the MLF-CL groups (*n* = 4/group) (Figure 6).

### 3.6. MLF-CL Exhibited Dose-Dependent In Vitro Cytotoxicity to L929 Murine Fibroblasts

**Figure 7:**
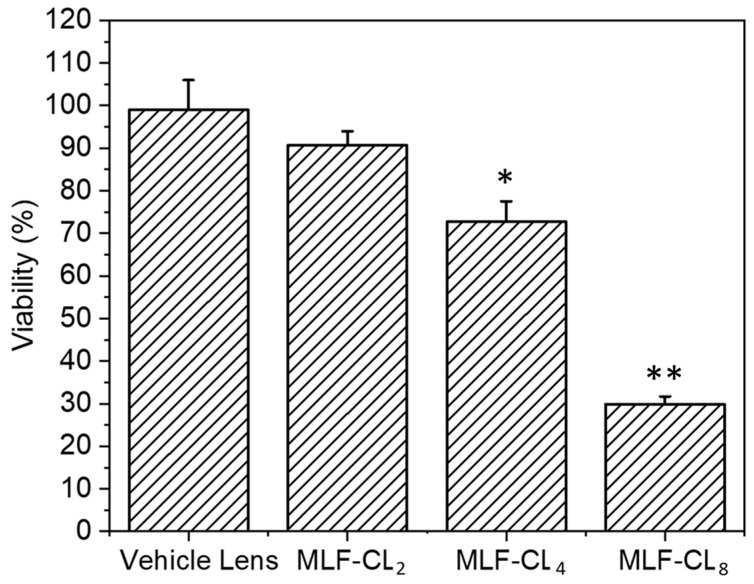
Cell viability of L929s with eluted media from miltefosine-eluting contact lenses (MLF-CLs). Results are expressed as percent viability of untreated L929s. Cell viability for MLF-CLs compared to vehicle lenses. * *p* = 0.017, ** *p* < 0.001 by Kruskal–Wallis Test with Bonferroni correction. *n* = 4.

Because miltefosine or MLF-CL leachables such as unreacted monomer, photoinitiator, or residual solvent could potentially cause toxicity, we tested the cytotoxicity of MLF-CLs by the minimum elution media method, where the extract from hydrated MLF-TCLs was exposed to L929 murine fibroblasts [45,46]. The L929 murine fibroblast cells are highly proliferative and frequently used in cytotoxicity testing to evaluate cellular viability and proliferation [47]. Results were compared to L929 cells not exposed to extract. Vehicle lenses showed no cytotoxicity (98.7 ± 7.3%). MLF-CLs showed viability that was inversely proportional to MLF content, with viabilities of 90.6 ± 3.3%, 72.7 ± 4.8%, and 29.8 ± 1.9% for MLF-CL_2,_ MLF-CL_4,_ and MLF-CL_8,_ respectively (Figure 7).

### 3.7. MLF-CLs Demonstrated Suitable Biocompatibility In Vivo

Potential risks with contact lens wear include corneal edema, cornea abrasions, and microbial keratitis and there is also the risk of local or systemic toxicity from topically delivered miltefosine. Normal New Zealand White (NZW) rabbits were used to assess biocompatibility as rabbits are the lowest order mammal that can wear a contact lens. In addition, NZW eye dimensions are comparable to those of a human [48]. MLF-CL_4_s were chosen to be evaluated in vivo as they had demonstrated suitable physicochemical properties similar to other commercial contact lenses while maintaining an acceptable cytotoxicity level. NZWs (*n* = 3, two females, one male) wore the MLF-CL_4_s continuously for three days. One eye showed erythema throughout the conjunctiva, on day 3. In the other two rabbits, no abnormalities were seen in daily observations during the lens wear period. No erythema, discharge, lid edema, or epithelial defects were noted on slit lamp exam after three days of continuous wear.

**Figure 8:**
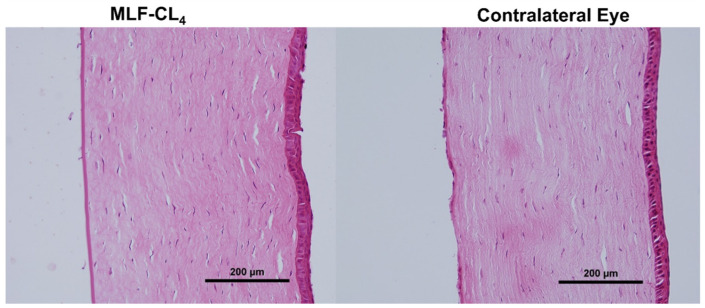
Hematoxylin and Eosin(H&E) stained corneas for New Zealand white rabbits (NZW) eyes wearing miltefosine-eluting contact lenses (MLF-CL_4_) compared to the contralateral eye (untreated).

Pathology revealed normal corneas and irises in all NZWs. No changes in the corneal epithelium, stromal lamellae, Descemet’s membrane, and endothelium were observed (Figure 8). Results were compared to the contralateral eyes of each rabbit, showing no difference between eyes that wore the MLF-CL and untreated eyes.

## 4. Discussion

In this work, we have encapsulated miltefosine in a contact lens hydrogel. This device provides sustained release of miltefosine for one week and may be of use in the treatment of AK. To our knowledge, this is the first report of a miltefosine sustained release system for the treatment of ocular disease.

PLGA was chosen due to its safety and excellent sustained drug release profiles [49]. PLGA is a component of numerous FDA- and European Medicine Agency (EMA)- approved drug delivery devices. The ratio of lactide to glycolide of 85:15 in PLGA was chosen because higher lactide content is associated with a decrease in degradation rate and drug release rate [49]. PLGA can degrade into its two monomeric constituents (lactic acid and glycolic acid) via hydrolysis reactions, which in turn can be easily metabolized via the Krebs cycle. Therefore, PLGA produces minimal systemic toxicity when used in biological applications [50], and showed no toxicity in our studies. The films were made by solvent casting and lathing, but other approaches could be employed, such as 3D printing of the film and producing contact lenses from molds [51].

In a miltefosine-loaded nanostructured lipid carriers release system [52], most drugs were released within a few hours. Miltefosine-loaded alginate nanoparticles sustained the release of miltefosine for 24 h [53]. In contrast, our MLF-CLs achieved a stable release rate over a period of 7 consecutive days. The sustained release of MLF-CLs may be attributed to the high partition coefficient (logP of 3.8) of miltefosine and interaction between drug and PLGA [54]. Miltefosine was miscible with PLGA in the drug-polymer film (Figure 1), indicating the interaction between drug and PLGA. Considering the hydrophobic properties of miltefosine and PLGA, this suggests drug-polymer interactions were due to this shared hydrophobicity with charge interactions as a secondary effect. The drug-polymer interaction can be investigated by Fourier-transform infrared spectroscopy (FTIR) and Differential Scanning Calorimeter (DSC) [55,56]. These results were consistent with our previous reports that PLGA films in contact lenses were able to sustain the release of hydrophobic drugs such as dexamethasone and latanoprost [35,41,57,58]. While AK often requires a treatment duration of weeks to months, extended wear of a single contact lens is limited to seven days to prevent complications from contact lens overwear, including corneal edema and microbial keratitis. In practice, a patient with AK could wear consecutive MLF-CLs—exchanged once weekly by an ophthalmologist—until AK is eradicated.

Previous studies have reported a minimum amebicidal concentration (MAC) of 16 µg/mL for clinical and environmental isolates [59,60,61]. The formulations MLF-CL_2,_ MLF-CL_4_ and MLF-CL_8_ released 24, 85 and 151 µg of miltefosine on the first day, respectively. The daily release rate thereafter ranged from 17-26 μg/day for MLF-CL_2,_ 24–42 μg/day for MLF-CL_4_, and ranged from 41-89 μg/day for MLF-CL_8_ (Figure 2B) after one week of testing, well above the MAC. Based on the design of contact lens drug delivery, the fluid exchange in the post-lens tear film that exists between the cornea and the contact lens has been shown to be slower than fluid exchange on the surface of the eye [62,63], which may improve the drug bioavailability compared to topical eye drops. In our previous study with a latanoprost contact lens, we found that contact lenses with drug-polymer films had high correlation coefficients between drug release in vitro and drug absorption in vivo [57]. We expect that the amount of miltefosine released by MLF-CLs in the eye may reach therapeutic amounts in vivo.

The light transmittance of central aperture was above 95% for all three formulation and was comparable to commercial lens, though the film-embedded part of the MLF-CL showed semitransparency (Figure 1). We also have the flexibility to increase the MLF-CL central aperture size to further increase visual performance.

Wettability and mechanical properties of the lenses are critical for the physiological compatibility, handling, durability, and comfort of wearing the lenses. In fact, oxygen diffusivity and water content are key parameters influencing the comfort and safety of a lens [36]. MLF-CLs showed similar water content compared to commercial methafilcon contact lenses of about 53% water uptake. At a range of 26–33°, MLF-CL wettability was also similar to commercial contact lenses. One of the limitations to our study is that we did not directly measure oxygen transmittance, which has been associated with wearing comfort.

The modulus range for the MLF-CLs we measured was between 210 kPa and 300 kPa. We found that MLF-CLs are softer than commercial contact lenses (490 kPa) as they showed a significantly lower modulus. This decrease in modulus is most likely the result of increased chain mobility in the contact lens hydrogel. Nonetheless, the modulus of MLF-CL is still within the acceptable range of values reported in the literature for the currently available contact lens materials (300 to 600 kPa) [64].

MLF-CL showed cell viability inversely proportional to dose: MLF-CL_2_ had the highest cell viability (91%) and MLF-CL_8_ had the lowest cell viability (36%). Given that the vehicle lens had cell viability of close to 100%, the decreased cell viability is solely associated with increased miltefosine dose. Nonetheless, MLF-CL_4_ had an acceptable level (73%) of cell survival by ISO standards [65]. In addition, Miltefosine was significantly less toxic than 0.02% (200 μg/mL) chlorhexidine, a standard concentration of chlorhexidine drops used to treat AK [66]. In our own studies, chlorhexidine showed significant toxicity at 0.005% (50 μg/mL), compared to miltefosine alone, which did not demonstrate cytotoxicity until 90 μg/mL (Figure S3). This is higher than the believed efficacious dose of 65.12 μg/mL. Previous in vitro studies on anti-AK drugs have shown low cell viability of 21% with 0.02% chlorhexidine (CHX) and 38% with both 0.02% polyhexamethylene biguanide (PHMB) and 0.01% (100 μg/mL) propamidine isethionate (PI). Given the favorable physicochemical properties of MLF-CL_4_, combined with minor in vivo toxicity, additional formulations can be investigated with changing miltefosine concentrations to reduce cytotoxicity. We also have the flexibility to adjust the MLF-CL central aperture size to achieve this purpose.

In the biocompatibility experiment for this study, one of three rabbits exhibited adverse effects after three days of wear. Miltefosine acts as an immunomodulator, activating Type 1 T-helper cells (Th1) which in turn increase the recipient’s inflammatory response. Cytokines, particularly interferon gamma and interleukin 12 have been observed to be elevated with oral miltefosine use [67]. However, pathology did not reveal any abnormalities in the cornea and iris, indicating the adverse effects of miltefosine were self-limiting. In clinical practice, the severe inflammation reaction is alleviated by prescribing oral and/or topical steroids [24], though the practice is often contested, as steroids can dampen the body’s natural immune response [68,69]. Topical steroids could be prescribed, or potentially include steroids in the MLF-CL. In vivo and in vitro correlation can be poor, which also supports testing in vivo biocompatibility. More research is needed (i.e., evaluating long-term outcomes of MLF-CL wear) to determine the appropriate course of action.

## 5. Conclusions

We incorporated the miltefosine-PLGA film into a commercially available hydrogel material to form a delivery system that can slowly release miltefosine. MLF-CL’s physical properties were not significantly different from commercial contact lenses in terms of light transmittance, water content, and wettability. This ocular drug delivery system provides a slow and steady release of miltefosine over the course of a week and has the safety profile to serve as a promising drug treatment platform for AK. More critical efforts are needed to develop an efficient and safe MLF-CL device that can be validated in animals and clinics. In accordance with the outcomes of this investigation, we envision that the MLF-CL_4_ lens is very suitable for further in vivo and clinical studies, as it shows comparable physicomechanical properties to the commercial contact lens, optimal biocompatibility, and an enhanced and sustained drug release over a period on one week.

## Figures and Tables

**Table 1:** MLF-CL formulations.

Formulation Name	Drug: Polymer Ratio
Commercial contact lens	N/A
MLF-CL_2_	2:25
MLF-CL_4_	4:25
MLF-CL_8_	8:25

## Data Availability

Not applicable.

## Supplementary Materials

The following supporting information can be downloaded at: https://www.mdpi.com/article/10.3390/pharmaceutics14122750/s1, Figure S1: The OCT image of hydrated MLF-CL; Figure S2: In vitro release profiles (actual cumulative release %) of MLF-CL and mathematical modeling (predicted release %) of the miltefosine release profiles. The in vitro release profiles of miltefosine from MLF-CL fit to the Korsmeyer-Peppas model. Data are shown as the means ± SD, n = 4 for each group; Figure S3: Cell viability of L929s subjected to different concentrations of (A) Miltefosine (µg/mL) and (B) Chlorhexidine free base drug (µg/mL); Table S1: Mathematical model examination for the miltefosine release from MLF-CL. R^2^-adjusted, Akaike Information Criterion (AIC) and the Model Selection Criterion (MSC) employed for model selection (n = 4). The best model possesses the highest value of R2-adjusted, the lowest AIC value and the largest MSC [34].
