# Supplementary material for: Topical Sustained Delivery of Miltefosine Via Drug-Eluting Contact Lenses to Treat Acanthamoeba Keratitis
Source: Pharmaceutics. 2022 Dec 8;14(12):2750. doi: 10.3390/pharmaceutics14122750 (PMC9781349; doi:10.3390/pharmaceutics14122750)
Supplement: Supplementary file 1 [file pharmaceutics-14-02750-s001.zip › pharmaceutics-1996009-supplementary.pdf]

pharmaceutics

MDPI

Supplementary Materials: Topical Sustained Delivery of Miltefosine Eluting Contact Lenses to Treat Acanthamoeba Keratitis

Lin Chen, Liangju Kuang, Amy E. Ross, Wissam Farhat, Nikolay Boychev, Sina Sharfi, Levi N. Kanu, Longqian Liu, Daniel S. Kohane and Joseph B. Ciolino

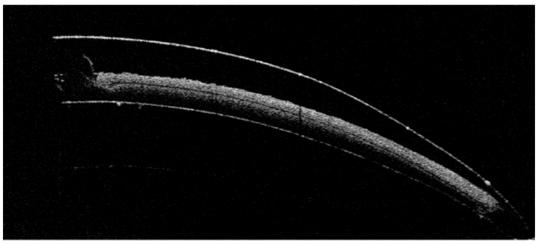

Figure S1. The OCT image of hydrated MLF-CL.

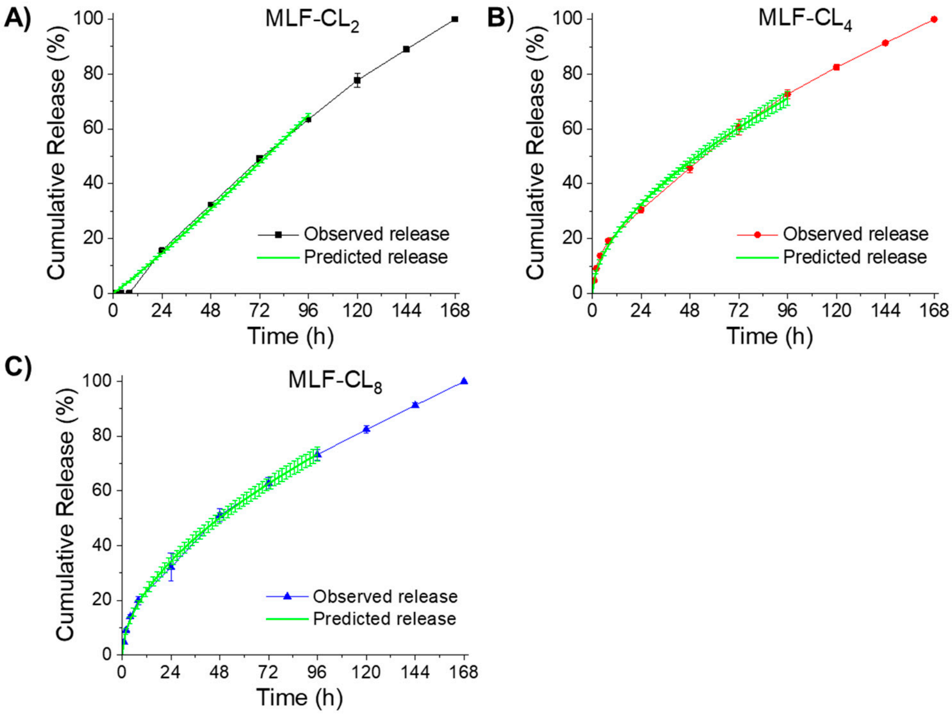

Figure S2. In vitro release profiles (actual cumulative release %) of MLF-CL and mathematical modeling (predicted release %) of the miltefosine release profiles. The in vitro release profiles of miltefosine from MLF-CL fit to the Korsmeyer-Peppas model. Data are shown as the means ± SD, n = 4 for each group.

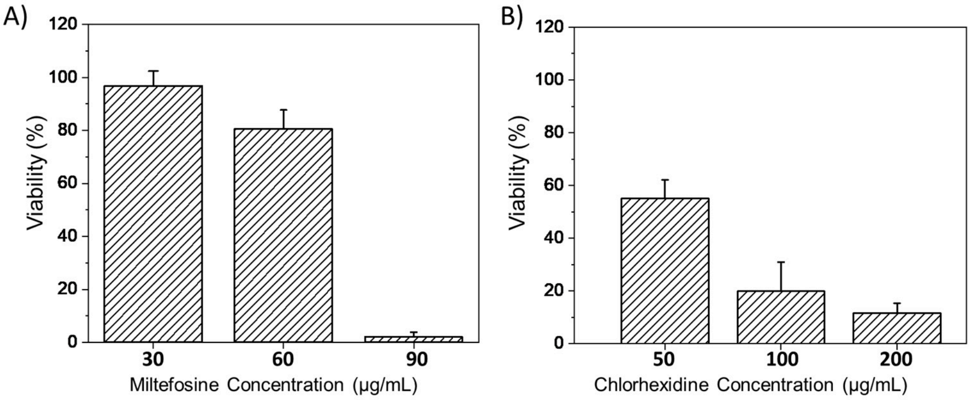

Figure S3. Cell viability of L929s subjected to different concentrations of A) Miltefosine (µg/mL) and B) Chlorhexidine free base drug (µg/mL).

Table S1. Mathematical model examination for the MLF release from MLF-CL. R^2-adjusted, Akaike Information Criterion (AIC), and the Model Selection Criterion (MSC) were employed for model selection (n=4). The best model possesses the highest value of R^2-adjusted, the lowest AIC value, and the largest MSC.

Model		$MLF\text{-}CL_2$		$MLF\text{-}CL_4$		$MLF\text{-}CL_8$	
		**Mean**	**SD**	**Mean**	**SD**	**Mean**	**SD**
**Zero-Order** $Q_t = Q_0 + K_0t$	K0 (%*h^{-1})	0.664	0.008	0.835	0.027	0.864	0.030
	R^2-adjusted	0.991	0.001	0.879	0.021	0.856	0.029
	AIC	31.568	0.750	52.177	1.124	54.086	1.623
	MSC	4.023	0.082	1.875	0.192	1.706	0.202
**First-Order** $\ln Q_t = \ln Q_0 + K_1t$	K (%*h^{-1})	0.009	0.000	0.014	0.001	0.015	0.001
	R^2-adjusted	0.971	0.001	0.950	0.013	0.952	0.015
	AIC	40.338	0.393	44.931	1.900	45.121	2.317
	MSC	2.927	0.061	2.781	0.315	2.826	0.359
**Higuchi model** $Q_t=K_H(t)^{1/2}$	K (%*$h^{-1/2}$)	5.244	0.075	7.025	0.215	7.306	0.273
	R^2-adjusted	0.845	0.004	0.989	0.004	0.990	0.010
	AIC	54.375	0.083	33.001	3.169	29.251	8.464
	MSC	1.172	0.020	4.271	0.361	4.810	1.054
**Korsmeyer-Peppas Model** $Q_t/Q_\infty=K_kt^n$	K (%*h^{-n})	0.480	0.054	5.382	0.501	6.004	0.825
	n	1.076	0.023	0.566	0.025	0.550	0.030
	R^2-adjusted	0.992	0.002	0.994	0.002	0.994	0.008
	AIC	32.0378	1.6398	28.371	2.319	25.720	8.262
	MSC	3.9646	0.192	4.850	0.367	5.252	1.060
